# Supplementary material for: Selected Correlates of Attitudes towards Rape Victims among Polish Medical Students
Source: Int J Environ Res Public Health. 2022 May 12;19(10):5896. doi: 10.3390/ijerph19105896 (PMC9141270; doi:10.3390/ijerph19105896)
Supplement: Supplementary file 1 [file ijerph-19-05896-s001.zip › ijerph-1687383-supplementary.pdf]

File S1.

Demographic information

1. Gender: a. Female, b. Male, 2. Age………….(years)

3. Matrimonial status: a. Maid/bachelor, b. Married, c. Divorced/Separated

4. Religious involvement: a. Strong, b. Average, c. Indifferent, d. Agnostic/Atheist

5. Place of residence: a. Big city, b. Small town, c. Countryside

6. Year of the study:

a. Undergraduate level (licentiate): 1st, 2nd, 3rd

b. Graduate level (master of arts): 1st, 2nd, 3rd

7. Field of study (major): …………………………………………………..

ATTITUDES TOWARD RAPE VICTIMS SCALE

Strongly disagree	Disagree mildly	Neutral	Agree midly	Strongly agree
**1**	**2**	**3**	**4**	**5**

1. A raped woman is a less desirable woman.	1	2	3	4	5
2. The extent of the woman's resistance should be the major factor in determining if a rape has occurred.	1	2	3	4	5
3. * A raped woman is usually an innocent victim.	1	2	3	4	5
4. Women often claim rape to protect their reputations.	1	2	3	4	5
5. * ″Good″ girls are as likely to be raped as “bad” girls.	1	2	3	4	5
6. Women who have had prior sexual relationships should not complain about rape.	1	2	3	4	5
7. * Women do not provoke rape by their appearance or behavior.	1	2	3	4	5
8. Intoxicated women are usually willing to have sex.	1	2	3	4	5
9. It would do some women good to be raped.	1	2	3	4	5
10. * Even women who feel guilty about engaging in premarital sex are not likely to claim rape falsely.	1	2	3	4	5
11. Most women secretly desire to be raped.	1	2	3	4	5
12. * Any female may be raped.	1	2	3	4	5
13. Women who are raped while accepting rides from strangers get what they deserve.	1	2	3	4	5
14. Many women invent rape stories if they learn they are pregnant.	1	2	3	4	5
15. * Men, not women, are responsible for rape.	1	2	3	4	5
16. A woman who goes out alone at night puts herself in a position to be raped.	1	2	3	4	5
17. Many women claim rape if they have consented to sexual relations but have changed their minds afterwards.	1	2	3	4	5
18. Accusations of rape by bar girls, dance hostesses and prostitutes should be viewed with suspicion.	1	2	3	4	5
19. * A woman should not blame herself for rape.	1	2	3	4	5
20. A healthy woman can successfully resist a rapist if she really tries.	1	2	3	4	5
21. Many women who report rape are lying because they are angry or want revenge on the accused.	1	2	3	4	5
22. * Women who wear short skirts or tight blouses are not inviting rape.	1	2	3	4	5
23. Women put themselves in situations in which they are likely to be sexually assaulted because they have an unconscious wish to be	1	2	3	4	5

raped.					
24. Sexually experienced women are not really damaged by rape.	1	2	3	4	5
25. In most cases when a woman was raped she deserved it.	1	2	3	4	5

* Items are reversed scored.

SOURCE: Ward, C. (1988). The Attitudes toward Rape Victims Scale: Construction, validation and cross-cultural applicability. Psychology of Women Quarterly, 12, 127-146.

SKALA POSTAW WOBEC OFIAR GWAŁTÓW (Wersja polska)

Instrukcja: Przeczytaj poniższe zdania i zaznacz w jakim stopniu zgadzasz się z ich treścią:

**Zdecydowanie nie zgadzam się**	**Częściowo nie zgadzam się**	**Nie mam zdania**	**Częściowo zgadzam się**	**Zdecydowanie zgadzam się**
**1**	**2**	**3**	**4**	**5**

1. Zgwałcona kobieta jest mniej pożądaną – atrakcyjną.	1	2	3	4	5
2. Wielkość oporu, jaki kobieta stawia, jest najważniejszym czynnikiem oceny czy gwałt miał miejsce.	1	2	3	4	5
3. Zgwałcona kobieta zwykle jest niewinną ofiarą.	1	2	3	4	5
4. Kobiety często zasłaniają się gwałtem w obronie swojej reputacji.	1	2	3	4	5
5. „Dobre" dziewczyny tak samo narażone są na gwałt jak „złe".	1	2	3	4	5
6. Kobiety, które wcześniej miały doświadczenia seksualne nie powinny narzekać na gwałt.	1	2	3	4	5
7. Kobiety nie prowokują do gwałtu swoim wyglądem lub zachowaniem	1	2	3	4	5
8. Pod wpływem środków psychoaktywnych kobiety są chętne do seksu.	1	2	3	4	5
9. Niektóre kobiety są bardziej predysponowane na bycie ofiarą gwałtu.	1	2	3	4	5
10. Nawet kobiety, które czują się winne z powodu stosunków przed-małżeńskich, nie są skłonne do oskarżania kogoś o gwałt bezpodstawnie.	1	2	3	4	5
11. Większość kobiet skrycie chciałaby być zgwałcona.	1	2	3	4	5
12. Każda kobieta może być zgwałcona.	1	2	3	4	5
13. Kobiety, które zostały zgwałcone gdyż akceptowały zaloty osób obcych, otrzymały to, na co zasłużyły.	1	2	3	4	5
14. Wiele kobiet zmyśla historię o gwałcie, kiedy zorientuje się, że	1	2	3	4	5

zaszły w ciążę.					
15. Nie kobiety, lecz mężczyźni odpowiedzialni są za gwałt.	1	2	3	4	5
16. Kobieta, która samotnie wychodzi w nocy, sama prowokuje gwałt.	1	2	3	4	5
17. Wiele kobiet oskarża kogoś o gwałt mimo, że zgodziły się na stosunek seksualny, jeśli później zmieniły zdanie.	1	2	3	4	5
18. Oskarżenia o gwałt stawiane przez kelnerki, tancerki i prostytutki należy traktować jako podejrzane.	1	2	3	4	5
19. Kobieta nie powinna oskarżać siebie o to, że została zgwałcona.	1	2	3	4	5
20. Zdrowa kobieta może z powodzeniem oprzeć się gwałcicielowi jeśli tego chce.	1	2	3	4	5
21. Wiele kobiet oskarżających kogoś o gwałt kłamie, gdyż czuje złość lub chce się zemścić na oskarżonym.	1	2	3	4	5
22. Kobiety noszące krótkie spódniczki lub obcisłe bluzki nie prowokują gwałtu.	1	2	3	4	5
23. Kobiety same stawiają siebie w sytuacjach narażających je na gwałt, gdyż kieruje nimi nieświadome pragnienie bycia zgwałconym.	1	2	3	4	5
24. Doświadczone seksualnie kobiety nie doznają szkody po gwałcie.	1	2	3	4	5
25. W większości przypadków zgwałcone kobiety zasłużyły sobie na to.	1	2	3	4	5
